# A novel telomerase substrate precursor rapidly induces telomere dysfunction in telomerase positive cancer cells but not telomerase silent normal cells

**DOI:** 10.18632/oncoscience.213

**Published:** 2015-08-22

**Authors:** Ilgen Mender, Sergei Gryaznov, Jerry W. Shay

**Affiliations:** ^1^ University of Texas Southwestern Medical Center, Department of Cell Biology, Dallas, TX, USA; ^2^ Hacettepe University, Faculty of Medicine, Department of Biochemistry, Ankara, Turkey; ^3^ AuraSense Therapeutics, Skokie, IL, USA; ^4^ Center of Excellence in Genomic Medicine Research, King Abdulaziz University, Jeddah, Kingdom of Saudi Arabia

**Keywords:** cancer, telomere shortening, telomere induced foci, 6-thioguanine, 6-thio-2′deoxyguanosine

## Abstract

Although telomerase is an almost universal target for cancer therapy, there has been no effective telomerase targeted inhibitor that has progressed to late stage human clinical trials. Recently, we reported that a telomerase-mediated telomere-disrupting compound, 6-thio-2′-deoxyguanosine (6-thio-dG), was very effective at targeting telomerase positive cancer cells while sparing telomerase silent normal cells. 6-thio-dG, a nucleoside analogue of the already-approved drug 6-thioguanine, is incorporated into telomeres by telomerase, resulting in disruption of the telomere-protecting shelterin complex. This disruption leads to Telomere dysfunction-Induced Foci (TIFs) formation and rapid cell death for the vast majority of cancer cells. Since most chemotherapies eventually fail due to drug acquired resistance, novel drugs such as 6-thio-dG, as a single first line agent or in the maintenance setting, may represent an effective new treatment for cancer patients.

## INTRODUCTION

At the end of linear chromosomes, repetitive sequence structures called telomeres are protected from being recognized as random DNA double strand breaks, which would otherwise activate DNA damage signaling responses. These sequences, (TTAGGG)n in mammals, are responsible for genomic stability by preventing recombination, end-end fusions/degradation and are also important in completing DNA replication during each cell cycle [1]. If telomeres become too short or “uncapped”, chromosome ends cannot be properly protected by the shelterin proteins that bind to telomeric repeats [2]. This results in formation of breakage-fusion-bridge cycles that lead to DNA double strand breaks and unstable multicentric chromosomes [3]. Under ideal culture conditions, growing normal cell populations eventually undergo senescence (growth arrest) due to progressive telomere shortening. A subset of cells can undergo cell death by bypassing senescence and entering crisis (where telomeres are even shorter compared to senescence). However, if rare cells escape crisis by loss of tumor suppressor gene functions, they acquire additional genomic changes. Importantly, these alterations in turn reactivate the ribonucleoprotein enzyme telomerase, which is present in ~90% of primary human tumors but not in most somatic tissue cells, with the exception of transiently proliferating stem-like cells. Therefore, telomerase is a highly attractive, almost universal, target for cancer therapy [4, 5].

While there have been many different approaches to directly or indirectly target telomerase, only Imetelstat (GRN163L) has progressed to late stage human clinical trials. One concern with Imetelstat is the development of hematological toxicities requiring drug holidays that enable telomere re-elongation. An effective inhibitor would ideally permit long-term, robust (>99%) telomerase inhibition or telomere dysfunction and rapid tumor shrinkage. Direct telomerase inhibitors in clinical trials do not show rapid tumor shrinkage not has robust telomerase inhibition been demonstrated. This is important since we have shown that only one percent of telomerase activity in cancer cells is sufficient to maintain the shortest telomeres and permit cells to continue to divide [6]. It is well established that telomerase preferentially elongates the shortest telomeres [7].

**Figure 1:**
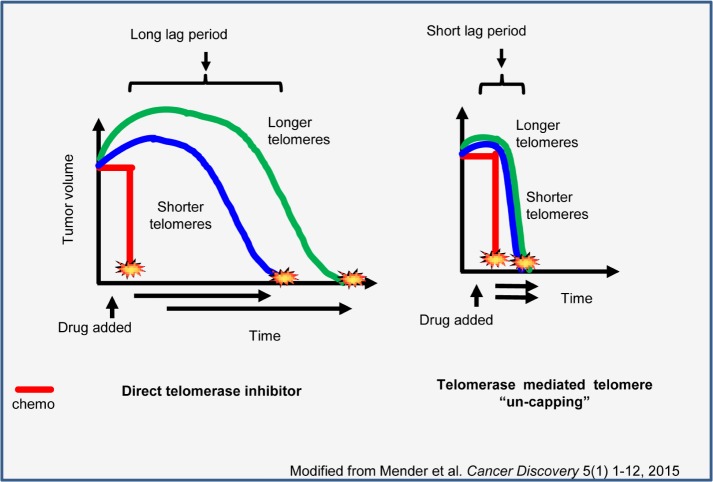
Comparison of two different approaches on telomerase targeted therapies Since direct telomerase inhibitors cause telomere shortening by inhibiting telomerase activity, their effect will be depend on initial telomere length of cancer cells. While these inhibitors will have a long time period to shorten telomeres and be effective on cancer cells with long telomeres, this time period will be shorter for the cancer cells with shorter telomeres. One of the main concerns for the direct telomerase inhibitor approach is that the long treatment period may cause side effects on patients and they will need to stop therapy, which will lead to telomere re-elongation. However, since a telomerase-mediated telomere “un-capping” approach is independent from initial telomere length we expect to have a much more rapid effect on cancer cells leading to tumor shrinkage.

Hence, we [8] and others [9] have sought to develop new approaches to targeting telomerase-expressing cancer cells. A base-modified nucleoside 6-thio-2′-deoxyguanosine (6-thio-dG) is an analogue of an already approved drug, 6-thioguanine. We reasoned that its 5′-triphosphate (formed *in situ* in cells) may be a telomerase-directed telomere uncapping compound. 6-thio-dG is rapidly converted to telomerase substrate 6-thio-2′-deoxyguanosine-5′-triphosphate (6-thio-dGTP) and consequently uses telomerase for its incorporation into telomeres. The guiding concept for initial proof-of-principle studies was that, since 6-thio-dG is converted rapidly into 6-thio-dGTP, it is potentially incorporated into both genomic DNA (by DNA polymerases) and telomeric DNA (by telomerase). We therefore predicted that 6-thio-dG would be both a more effective agent compared to 6-thioguanine and also induce cancer cell killing much more rapidly than a “classic” telomerase inhibitor. Once 6-thio-dG is incorporated into telomeres, the telomere sequence TTAGGG is modified at guanine bases, resulting in uncapping of telomeric DNA and likely loss of recognition and dissociation of the shelterin proteins from the *de-novo* formed modified telomeres. This leads to Telomere dysfunction Induced Foci (TIF) formation and rapid growth arrest or cell death of telomerase-positive cells. This relatively fast anti-cancer effect of 6-thio-dG has an important advantage compared to other direct telomerase inhibition approaches. Long and frequent treatment cycles of direct telomerase inhibition-based therapies can cause side effects, as evidenced in some anti-telomerase clinical trials [10]. Importantly, the long lag period from initiation of treatment until cell death is dependent on initial cancer cell telomere length. Thus, patients with relatively long telomeres would require longer treatment periods and may be therapeutically disadvantaged by a direct telomerase inhibitor that is unlikely to stop the growth of the tumor cells before tumor burden is so extensive that there is no overall patient survival advantage. However, 6-thio-dG, a telomerase-mediated telomere uncapping agent, exerts a much more rapid effect, with a markedly reduced lag period. Importantly, this effect appears to be independent from initial cancer cell telomere lengths, as anticipated based on the compound's mechanism of action (Figure 1). In our studies, we have also demonstrated 6-thio-dG-induced telomere dysfunction in hTERT expressing cancer cells, as well as rapid and progressive telomere shortening in cancer cells that survive the initial treatment. Importantly, 6-thio-dG has minimal effects on normal human telomerase-silent cells. In addition, mice that have been treated up to one month with therapeutic concentrations of 6-thio-dG do not lose weight and hematological, liver and kidney functions remain in the normal range. At the same time, in normal human fibroblasts with transfected with hTERT (e.g. ectopically introduced telomerase activity), 6-thio-dG induces TIFs and cell death, demonstrating a direct relationship between telomerase, 6-thio-dG and induction of TIFs. These results are independent from direct inhibition of telomerase activity in vitro, since 6-thio-dG does not inhibit telomerase activity in cancer cells, but instead uses telomerase's preferential incorporation of 6-thio-dG into telomeres to induce telomere uncapping. Moreover, while 6-thio-dG treatment causes cell death for the vast majority of cancer cells tested, normal fibroblasts and normal human colonic epithelial cells were largely unaffected. These results represent an attractive chemotherapeutic approach to primarily target telomerase expressing cancer cells, sparing normal cells. Indeed, in a small percent of cancer types that do not engage telomerase, these ALT (Alternative Lengthening of Telomeres) cells were also affected by 6-thio-dG, showing that general DNA damage of 6-thio-dG can lead to cell death, which, however, takes place at higher concentrations compared to effects on telomerase positive cells. Additionally, lung cancer cell based xenograft model studies showed dramatic tumor reduction, as well as telomere dysfunction in vivo induced by 6-thio-dG treatment.

In summary, application of 6-thio-dG, with its proposed telomere-targeting mechanism of action, appears to be a promising anti-cancer treatment approach. As with any chemotherapy treatment, 6-thio-dG resistance mechanisms are expected to emerge. Understanding these resistance mechanisms may lead to more personalized based therapeutic regimens. In preliminary studies, we have tested a series of commonly-used multi-drug resistant cell lines and found 6-thio-dG to be active and effective in a significant fraction of these cell lines. Thus, 6-thio-dG may be effective in the maintenance setting after first line chemotherapy, with treatment yielding long-term durable responses for cancer patients.
